# Emergency total extracorporeal life support versus standard advanced cardiac life support with rescue extracorporeal membrane oxygenation for refractory out-of-hospital cardiac arrest: protocol for the ECLS-OHCA randomized trial

**DOI:** 10.1186/s13049-026-01557-w

**Published:** 2026-02-03

**Authors:** Hsun-Yi Fu, Yuan-Hung Liu, Shao-Jung Li, Heng-Chia Chang, Chun-Chieh Liu, Chi-Ming Lee, Chunn-Yao Huang, Chin-Wang Hsu, Chow-In Ko, Yi-Chih Wang, Wei-Tien Chang, Ta-Jung Wang, Jiann-Ruey Ong, Jen-Tang Sun, Jer-Shen Chen, Chan-Yang Hsu, Shih-Jung Jang, Yu-Long Chen, Yih-Hsin Lin, Chen-Yen Chien, Yu-Chung Kung, Tzong-Luen Wang, Hao-Chun Yu, Chaun-Chih Hsu, Chun-Chien Chao, Chih-Wei Chen, Chien-Yi Hsu, Shih-Chang Hsu, Te-I. Chang, Jong-Shiuan Yeh, Min-Shan Tsai, Ling-Yi Wei, Heng-Wen Chou, Chih-Hsien Wang, Chin-Hao Chang, Chi-Ling Chen, Yong-Kwang Tu, Jiunn-Lee Lin, Yih-Sharng Chen

**Affiliations:** 1 Department of Surgery, College of Medicine, National Taiwan University Hospital, National Taiwan University, Cardiovascular Division, Taipei, Taiwan; 2 Far Eastern Memorial Hospital, New Taipei City, Taiwan; 3 Depatment of Internal Medicine, Taipei Medical University-Shuang Ho Hospital, Ministry of Health and Welfare, New Taipei City, Cardiology, Taiwan; 4 Taipei Tzu Chi Hospital, Buddhist Tzu Chi Medical Foundation, New Taipei City, Taiwan; 5 MacKay Memorial Hospital (Tan-Sui), New Taipei City, Taiwan; 6 Fu Jen Catholic University Hospital, Fu Jen Catholic University, New Taipei City, Taiwan; 7 Taipei Medical University Hospital, Taipei, Taiwan; 8 Taipei Municipal WangFang Hospital, Taipei, Taiwan; 9 Department of Emergency, School of Medicine, College of Medicine, College of Medicine, National Taiwan University Hospital, National Taiwan University, Taipei, Taiwan; 10 Department of Internal Medicine, Cardiology Division, School of Medicine, College of Medicine, College of Medicine, National Taiwan University Hospital, National Taiwan University, Taipei, Taiwan; 11 Graduate Institute of Clinical Medicine, College of Medicine, National Taiwan University, Taipei, Taiwan

**Keywords:** Extracorporeal life support, Extracorporeal membrane oxygenation, Out-of-hospital cardiac arrest, Randomized clinical trial, Refractory cardiac arrest, Shockable rhythm

## Abstract

**Background:**

Out-of-hospital cardiac arrest (OHCA) is a time-sensitive emergency associated with high mortality and substantial risk of neurological disability. Extracorporeal cardiopulmonary resuscitation (ECPR) using extracorporeal membrane oxygenation (ECMO) has been introduced for refractory cardiac arrest, but recent randomized controlled trials have reported conflicting results, and the benefit of ECPR implemented according to rigorously timed protocols remains uncertain.

**Aim:**

This trial evaluates whether emergency total extracorporeal life support (ECLS), with emphasis on expedited cannulation and active left ventricular (LV) unloading, improves 30-day survival with favourable neurological outcome compared with standard advanced cardiac life support (ACLS) with rescue ECLS in adults with refractory shockable out-of-hospital cardiac arrest.

**Design, setting, and participants:**

This is a prospective, investigator-initiated, multicenter, open-label randomized clinical trial conducted in eight experienced extracorporeal membrane oxygenation (ECMO) centers in the Taipei metropolitan area. Eligible adults aged 18–75 years with witnessed refractory shockable OHCA (ventricular fibrillation or pulseless ventricular tachycardia) who received bystander cardiopulmonary resuscitation (CPR) are randomized 1:1 on emergency department (ED) arrival.

**Interventions:**

Participants are assigned to: (1) emergency total ECLS, with initiation as soon as possible after randomization and within 60 min of the emergency call, or (2) standard ACLS with rescue ECLS, in which at least 15 min of standard ACLS is provided in the ED before ECLS is considered. Both groups receive immediate coronary angiography and percutaneous coronary intervention (PCI) when indicated, and active LV unloading is recommended according to prespecified criteria.

**Primary endpoint:**

Survival at 30 days with favorable neurological status, defined as a Cerebral Performance Category score of 1 or 2.

**Conclusion:**

The ECLS-OHCA trial will provide complementary randomized evidence on the impact of expedited time-to-flow and protocolized LV unloading on neurological outcomes in refractory shockable OHCA and will explore the cost-effectiveness of this strategy.

**Trial Registration:**

ClinicalTrials.gov: NCT06692075.

**Supplementary Information:**

The online version contains supplementary material available at 10.1186/s13049-026-01557-w.

## Introduction

### Global burden of out-of-hospital cardiac arrest

Out-of-hospital cardiac arrest (OHCA) is a global public health burden, characterized by the high mortality, the potential for long-term disability, and the strain on healthcare systems. The incidence of emergency medical services (EMS)-treated OHCA has been reported as 40.6 per 100,000 person-years in Europe, 47.3 in North America, 45.9 in Asia, and 51.1 in Australia [1]. Hospital survival after OHCA varies substantially by region, and a global survival rate is estimated to be around 8 to 10% [2].

OHCA is a highly time-sensitive medical emergency whose outcome is heavily impacted by prompt resuscitation and intervention before irreversible organ damage develops. Early recognition of OHCA and activation of the emergency response system, and immediate initiation of cardiopulmonary resuscitation (CPR) result in a doubling or even tripling of survival rates of OHCA [3]. For individuals experiencing OHCA with shockable rhythms (e.g., ventricular fibrillation (VF) and pulseless ventricular tachycardia (pVT)), early defibrillation is the key action to further improve the chances of survival [4]. According to the International Liaison Committee on Resuscitation (ILCOR) survey in 2025, survival to hospital discharge or 30-day survival after EMS-treated OHCA and bystander-witnessed shockable OHCA ranged from 3.1% to 20.4% and from 11.7% to 47.4%, respectively [1].

### Extracorporeal cardiopulmonary resuscitation for refractory OHCA

Refractory cardiac arrest, defined as the failure to achieve sustained return of spontaneous circulation (ROSC) despite prolonged CPR efforts or multiple defibrillation attempts, is associated with an even worse prognosis [5]. It is estimated that greater than 70% of patients with shockable OHCA are refractory to conventional CPR and remain pulseless on arrival at the emergency department (ED) [6]. Extracorporeal cardiopulmonary resuscitation (ECPR) is the application of extracorporeal membrane oxygenation (ECMO) or extracorporeal life support (ECLS) in patients with refractory cardiac arrest, aiming to immediately reconstitute the cardiopulmonary function outside patients’ bodies and thus buy time for the delivery of interventions necessary to regain an adequate native circulation [7]. As the most efficient approach of resuscitation for refractory cardiac arrest, the number of ECPR runs has increased tenfold over the last decades [8]. Multiple observational studies have supported the use of ECPR in refractory OHCA [9]. Encouragingly, in expert institutions with highly dedicated and well-trained ECPR teams, a > 40% functionally intact survival of refractory OHCA could be achieved [9, 10]. However, recent randomized controlled trials (RCT) have reported seemingly inconsistent results of ECPR for refractory OHCA [11–13]. Latest update on Adult Advanced Cardiovascular Life Support (ACLS) from the ILCOR Consensus on Science With Treatment Recommendations recommendation assigns a IIa recommendation for use of ECPR in patients with cardiac arrest refractory to standard ACLS [14].

While first responders’ actions are critical to grab the chance of survival in patients with OHCA, studies have shown that the probability of survival with good functional recovery declines rapidly with each minute of CPR surpassing the effective duration, and that repeating the same therapies that did not work during this window did not yield significant survival benefits [15]. To boost the survival without severe impairment in patients with refractory OHCA, efficient strategies to minimize the low-flow duration should be considered. According to the Extracorporeal Life Support Organization guideline, ECPR should be considered after 10–15 min of unsuccessful conventional resuscitation efforts [7]. Observational data suggested that time to ECMO inversely correlated with neurologic outcomes in patients receiving ECPR [16]. In view of the inherent drawbacks of time elapsed in prehospital resuscitation in OHCA, as reflected in three published RCTs (the average interval from emergency calls to ECMO initiation was 59 min, 62 min, and 74 min, respectively), we believe that the benefits of ECPR for refractory OHCA should be investigated in the context of more timely application [17]. The similar result from retrospective data in ECPR also demonstrated 60 min between arrest-to-ECMO was an important factor for better neurological outcome [18].

To add to the evidence on the effectiveness of ECPR for patients with refractory OHCA with longer duration of low-flow time, we propose the present prospective randomized open label clinical trial. The centers are all experienced centers in performing ECPR and coronary intervention and located in the Taipei metropolitan area.

### Knowledge gap

The three recent RCTs [11–13] (ARREST, Prague OHCA, INCEPTION) showed conflicting results. The real answer in this field is debatable. The key limitations of previous trials included single center or limited cases number, or trial in multiple centers with different various experience, lack of standardized post-cardiac arrest care protocols, and absence of systematic LV unloading strategies.

Our trial specifically addresses these gaps by: (1) expedited ECPR with target ≤ 60 min from emergency call to ER, and a 15-min standard ACLS in expert location/team to evaluate standard ACLS in the ECPR setting. (2) standardized post-cardiac arrest care including early coronary angiography, and (3) protocolized active LV unloading.

Our hypothesis is that early initiation of ECLS, compared with conventional ACLS with rescue ECLS, could increase 30-day neurologically favorable survival of patients experiencing refractory OHCA presented with initial shockable rhythm.

### Novelty

Our protocol is designed to address this by incorporating active LV unloading strategies under prespecified criteria (see below). Early active LV unloading during ECMO support improves survival in patients with cardiogenic shock, and concomitant LV unloading has been associated with favorable outcomes in refractory cardiac arrest. We incorporate the concept into the ECPR to validate its efficacy. The estimated call to ECMO perfusion may be expected longer than previous studies since we allowed emergency call to ED within 60 min.

## Study design

This is an investigator-initiated, multicenter, open-label, interventional randomized clinical trial. The responsible investigator is the **Taipei Metro Out-of-Hospital Cardiac Arrest Total Care** *Consortium.*

The trial has been registered on ClinicalTrials.gov (NCT06692075).

### EMS in Taipei metropolitan area

In response to emergency calls for OHCA events, the Taipei Metro EMS system proceeds with instant online dispatcher-assisted CPR by witnessed bystanders with or without a public automatic external defibrillator (AED) at the same time as EMS team dispatching. On arrival at the scene, the EMS team will perform ACLS, including high-quality chest compression, basic or advanced airway management, intravenous or intraosseous access establishment, or automated chest compression by the Lund University Cardiopulmonary Assist System, and AED defibrillations if needed, followed by intra-arrest escorted transportation with continuous telecommunication to emergency service of the Taipei Metro major disease emergency responsible hospitals in the Taipei Metro OHCA Total Care Consortium.

The Taipei metropolitan area has a densely distributed healthcare system. Based on recent data, the mean EMS response time (from emergency call to arrival at scene) is 6.4 ± 3.3 min, and the mean EMS transport time (from scene to ED arrival) is 4.5 ± 2.8 minutes [19]. Since the some buildings without elevator and the EMS had standard procedures to be performed on scene, and the area of New Taipei City is 7 times than Taipei City, we estimate the mean time from call to ED arrival approximately 40–60 min.

### Enrollment and randomization of trial population

**Fig. 1:**
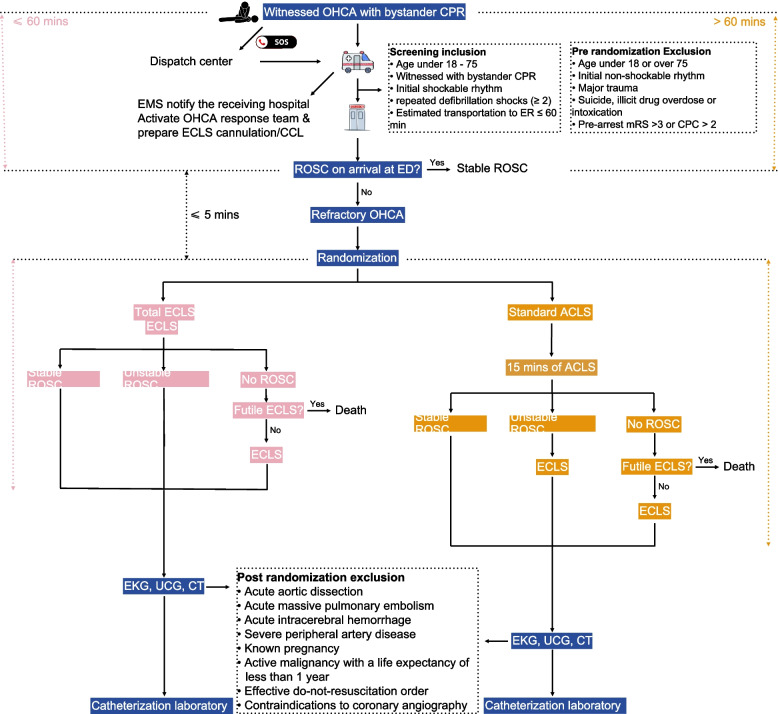
Study flowchart. Before arriving at ED, the eligibility of OHCA patients will be evaluated by the OHCA response team of the receiving hospital, based on on-scene information provided by EMS. For eligible OHCA patients, randomization will be completed within 5 min of arrival at ED. After CT imaging, patients of first enrollment will be checked again if any exclusion criteria are met. Only patients who received early coronary angiography and/or intervention will be included in the final analysis. ACLS: advanced cardiovascular life support; CCL: cardiac catheterization laboratory; CPC: cerebral performance category; CPR: cardiopulmonary resuscitation; CT: computed tomography; ECLS: extracorporeal life support; ED: emergency department; EKG: electrocardiography; EMS: emergency medical services; mRS: modified Rankin scale; OHCA: out-of-hospital cardiac arrest; ROSC: return of spontaneous circulation; ECG: echocardiography

Refractory OHCA is defined as cardiac arrest in patients transported to hospital with ongoing CPR. Upon receiving the EMS’s referral of OHCA patients, the OHCA response team, composed of emergency physicians, interventional cardiologists, and cardiac surgeons, will evaluate the eligibility of patients based on on-scene information. The inclusion and exclusion criteria are described in Table 1. When the patient arrives at the ED, a rapid screening is performed to reassure his/her eligibility. All eligible patients will be randomized into the emergency total extracorporeal life support (total ECLS) group and the standard advanced cardiac life support with rescue ECMO (**standard ACLS**) group (Fig. 1).

**Table 1:** PreInclusion and exclusion criteria

Screening inclusion criteria	Pre randomization Exclusion	Post-randomization exclusions criteria
• Aged 18–75 years old	• Age under 18 or over 75	• Age < 18 years or > 75 years old
• Witnessed cardiac arrest with bystander cardiopulmonary resuscitation	• Major trauma	• Initial non-shockable rhythm, i.e. pulseless electrical activity or asystole
• Initial shockable rhythm, i.e. ventricular fibrillation or pulseless ventricular tachycardia	• Initial non-shockable rhythm	• Acute aortic dissection
• Need for repeated defibrillation shocks (> 2) by external defibrillator	• Major trauma	• Acute massive pulmonary embolism
• Estimated transportation time from activation of the emergency response system to arrival at emergency department of participant institutions less than 60 min	• Suicide, illicit drug overdose or intoxication	• Acute intracerebral hemorrhage
	• Pre-arrest mRS > 3 or CPC > 2	• Major trauma due to blunt, penetrating or burn injury
	• Unwitnessed CPR	• Severe peripheral artery disease
		• Known pregnancy
		• Suicide, illicit drug overdose or intoxication
		• Known pre-arrest mRS > 3 or CPC > 2
		• Active malignancy with a life expectancy of less than 1 year
		• Effective DNR order
		• Absolute contraindications to emergency coronary angiography, including known anaphylactic reaction to angiographic contrast media, acute bleeding

CPC, cerebral performance category; DNR, do-not-resuscitation; mRS, modified Rankin score

Randomization is performed immediately upon ED arrival (target: within 5 min) for patients meeting inclusion criteria and experiencing refractory cardiac arrest (defined as ongoing CPR on arrival). Patients with stable ROSC before randomization (defined as sustained pulse with systolic BP ≥ 90 mmHg without ongoing chest compressions for ≥ 2 min) are not randomized and proceed directly to coronary angiography.

Patients with unstable ROSC defined as: (1) return of pulse but requiring ongoing chest compressions, (2) recurrent VF/VT requiring repeated defibrillation, (3) persistent profound hypotension with systolic BP < 90 mmHg or mean BP < 60 mmHg despite vasopressor support, or (4) intermittent epinephrine injection to maintain the heart beating are eligible for randomization."

A computerized randomization list is provided by the National Taiwan University Hospital Clinical Trial Center by a software app (REDCAP). The randomization is in 1:1 ratio, stratified by participant hospitals, with a permuted block of 4. In-hospital randomization is conducted by OHCA response team coordinators immediately after the eligible patients arrive at ED. The randomization codes will be maintained by the National Taiwan University Hospital Clinical Trial Center and monitored by the Data Safety Monitoring Committee. The EMS teams and intensivists in the cardiac intensive care unit (ICU) will be blinded to the study group assignment and follow the institutional standards for post-cardiac arrest care.

### Futility criteria

We applied the futility criteria based on ELSO guideline, ARREST trial and German consensus [8, 11, 20], and these criteria must be met in combination ($\ge$ 2) to consider futility. The criteria include: 1. End-tidal CO2 < 10 mmHg, 2. PaO2 < 50 mmHg despite maximal ventilation, 3. Serum lactate > 20 mmol/L.

#### Post resuscitation protocol

After initial stabilization at ED, electrocardiography and echocardiography would be arranged to identify cardiac causes for OHCA. Computed tomography would be arranged (either before or after cath intervention) to exclude non cardiac causes of OHCA or contraindication (Table 1). All patients without contraindications for coronary intervention will then be transferred to the cardiac catheterization laboratory (CCL) for coronary angiography. With successful emergent management, patients are admitted to the cardiac ICU for continued post-cardiac arrest care.

#### Intervention—ECMO initiation

After randomization, if the enrolled patient has stable ROSC before initiating ECMO, he/she will be transferred to the cardiac catheterization laboratory (CCL) without ECMO support. The indications for ECMO include no ROSC or unstable ROSC (persistent or recurrent VF or pVT, profound cardiogenic shock with systolic blood pressure (BP) < 90 mmHg or mean BP < 60 mmHg despite intense inotropic support (bolus of epinephrine > 3 bolus), or persistent hypoxia with arterial oxygen saturation (SaO2) < 90% despite maximal ventilation support). The femoral artery and vein access will be cannulated under ultrasound guidance. ECMO would be deemed futile if more than one discontinuation criterion suggesting ineffective resuscitation (end-tidal carbon dioxide < 10 mmHg, partial pressure of oxygen in arterial blood < 50 mmHg or oxygen saturation < 85%, or serum lactate > 20 mmol/L) has been met.

However, if unstable ROSC persists as defined above, ECMO is initiated according to the randomization arm protocol. For the emergency total ECLS group (Fig. 1), the veno-arterial ECMO (VA-ECMO) will be initiated within 15 min after randomization. For standard ACLS with the recue ECMO group, patients will receive 15 min of ACLS at ED first. If there is no stable ROSC, the futility of ECLS would be reevaluated. Therefore, the recue VA-ECMO should be initiated after 15 min since randomization.

#### LV unloading in ECMO-supported patients

During ECMO support, active left ventricular (LV) unloading by balloon atrial septostomy, direct left atrial venoarterial (LAVA) cannulation, Impella®, percutaneous LV/pulmonary artery venting, would be considered when LV global akinesia, absence of aortic valve opening, or spontaneous echocardiographic contrast within LV is demonstrated by echocardiography, or frothy sputum with pulmonary congestion is present, or pulse pressure < 10 mmHg, or elevated left ventricular end-diastolic pressure $\ge$ 30 mmHg (Table 2).

**Table 2:** Criteria and indications for active left ventricular (LV) unloading

1. Persistent refractory VF/VT even after ECMO
2. Non-opening aortic valve
3. Pulse pressure < 10 mmHg
4. Severe LV dilatation/global hypokinesia
5. Severe pulmonary edema with frothy sputum
6. LVEDP $$\ge$$ 30 mmHg
7. “Smoke” image in LV, spontaneous echocardiographic contrast

ECMO: extracorporeal membrane oxygenation; LV: left ventricle; LVEDP: left ventricular end-diastolic pressure; VF: ventricular fibrillation, VT: ventricular tachycardia

### Post-cardiac arrest care protocols

#### Targeted Temperature Management (TTM)

Target temperature of 33–36 °C for 24 h, followed by controlled rewarming at 0.25–0.5 °C per hour for those without rescue ECMO. For the ECMO-supported victim, prevention of hyperthermia (> 37 °C) is warranted, intentional hypothermia is not mandatory according to the policy for individual centers. Prevention of shivering with neuromuscular blockade was administrated if needed. When the patient becomes agitated after resuscitation, the midazolam and analgesia with fentanyl will give for sedation.

#### ECMO management (in rescue ECMO)

The target flow rate of ECMO was kept around 60–80 mL/kg/min, and the anticoagulation: unfractionated heparin targeting a PTT 50–70 s. Complication of ECMO (limb ischemia, bleeding, thrombosis, etc.) was carefully monitored, recorded and reported in the case report form (CRF) [8].

#### Hemodynamic targets

The mean arterial pressure are maintained around 65–75 mmHg, and the central venous oxygen saturation: > 70%. The lactate level was monitored as regular fashion.

#### Mechanical ventilation

We apply the lung-protective ventilation with tidal volume 6–8 mL/kg ideal body weight, the plateau pressure < 30 cmH_2_O, titrated to maintain saturation over 90%.

#### Neuroprognostication

Neuroprognostication will be determined at least 5–7 days after Return of Spontaneous Circulation (ROSC) for patients who received Targeted Temperature Management (TTM). This timing ensures sufficient clearance of sedatives and resolution of potential drug effects or reversible physiological abnormalities before any prognosis is established. The assessment will occur approximately 72 h after the patient has reached normothermia following the TTM period. This timing ensures sufficient clearance of sedatives and resolution of potential drug effects or reversible physiological abnormalities before any prognosis is established.

The use of sedatives may affect the evaluation of the assessment of neurological status. We prioritize protocol to minimize the lingering effect of sedation. The clinical practice is discontinuation of sedation 72 h before assessment.

Prognostication utilizes a multi-modal approach incorporating multiple clinical and objective tests, such as neuroimages (CT and/or MRI) and biomarkers [21]. Not a single information determine the decision-making.

### Withdrawal-of-Care (WLST)

#### Criteria

Decisions regarding WLST are made only after the **c**ompletion of the multi-modal neuroprognostication protocol confirms an irreversible, devastating neurological prognosis.

Standardized Criteria: Criteria for poor prognosis leading to potential WLST are standardized across all centers and must include conclusive evidence from 2 to 3 modalities indicating a near-zero probability of functional recovery. These criteria include: (1)Persistent coma without brainstem reflexes on neurological exam, (2) Diffuse, severe anoxic injury on MRI/CT, (3)Burst suppression or status epilepticus refractory to treatment on EEG.

Decision made by multidisciplinary team after comprehensive neuroprognostication, and consultation about the goal of care under Shared decision making (SDM) process [21].

#### Follow-up

**Fig. 2:**
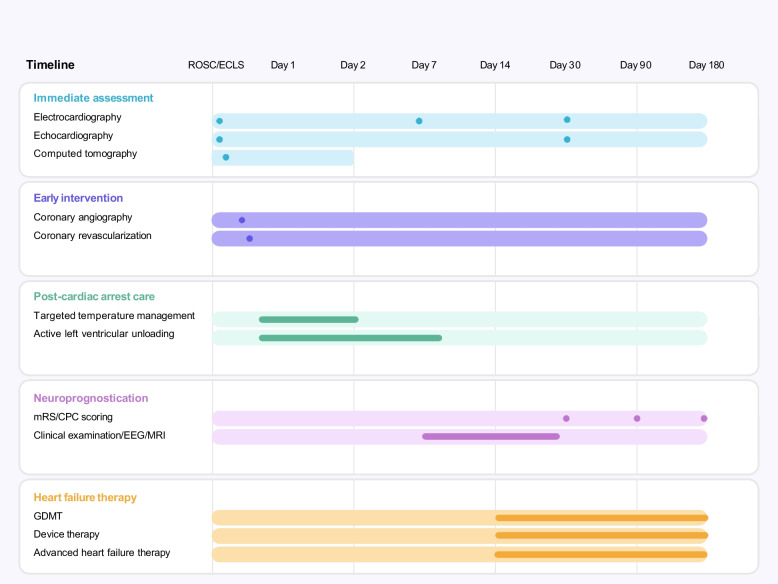
Timeline of post-resuscitation management and follow-up. This schematic outline illustrates the temporal progression of key clinical interventions following ROSC or initiation of ECLS. Early actions such as coronary angiography, targeted temperature management, and LV unloading might occur within the first days. Intermediate evaluations, including clinical examination, and EEG or MRI in patients who remain comatose, are emphasized at least 5–7 days after ROSC. Clinical examination will be assessed at 30 days, 90 days and 180 days after cardiac arrest. Long-term strategies incorporating GDMT, device therapy or advanced therapy might be considered for survivors with heart failure. CPC: cerebral performance category; ECLS: extracorporeal life support; EEG: electroencephalogram; GDMT: guideline-directed medical therapy; LV: left ventricular; MRI: magnetic resonance imaging; mRS: modified Rankin scale; ROSC: return of spontaneous circulation

All enrolled patients will be followed at hospital discharge and 1 month, 3 months, and 6 months after discharge. Survival status, neurological and functional recovery, readmission, and causes of death or readmission will be assessed via periodic interview or scheduled outpatient appointment (Fig. 2).

#### Study endpoints

The primary endpoint is defined as 30-day survival with Cerebral Performance Category (CPC) score of 1 or 2). The CPC score will be assessed by the designated neurologists at each participant hospital, who are blinded to the intervention received. Secondary endpoints include favorable neurological status (modified Rankin scale (mRS) < 3, crude survival and neurologically favorable survival at discharge, 3 months, and 6 months; duration of mechanical ventilation; duration of stay in ICU; duration of hospitalization; causes of hospital mortality and late mortality; and health care costs. The incidence of serious adverse events related to CPR, ECMO, coronary angiography, and LV unloading will be documented as safety endpoints.

For patients still hospitalized at 30 days, bedside assessment is performed. For discharged patients, assessment is conducted during scheduled follow-up clinic visits or via structured telephone interview using validated instruments.

### Statistical analysis

#### Sample size rationale

The trial is designed to demonstrate a treatment effect on the primary endpoint. Accepting a power of 80% and a level of significance of 5%, the sample size is calculated using a two-sided chi-square test. In reference to published multicenter trials, we estimate a 30-day neurologically favorable survival of 25% and 12% in the emergency total ECLS group and the standard ACLS with rescue ECMO group, respectively. Assuming a 10% dropout rate, we plan to enroll 186 participants per arm (roughly 200). No interim analysis is planned. If interim analysis is deemed necessary determined by Consortium committee, we will conduct one interim analysis 1 year after enrollment, and the statistical testing will be adjusted to this accordingly. The flowchart of intention to treatment and per protocol are attached in supplement.

All patients enrolled in the study will be analyzed primarily on an intention-to-treat basis. Continuous variables will be reported as mean ± standard deviation or median with interquartile range. Categorical variables will be presented as frequency. A logistic regression model with the stratification variable (center) will be conducted to evaluate the primary efficacy endpoint by treatment group. Odds ratios are to be calculated as effect estimates with 95% confidence intervals. Patients whose primary outcome cannot be assessed will be regarded as failures.

Per-protocol analysis, which only includes patients who adhere to the allocated protocol, will be performed to minimize the impact of non-adherence or deviations from the strict protocol.

For patients randomized to the total ECLS group, if ECPR is not required due to stable ROSC at ED or ECMO is initiated after 20 min since arriving at ED or after 60 min since emergency calls, they would be excluded from per-protocol analysis. Similarly, for patients randomized to the standard ACLS with rescue ECMO group, if ECMO is initiated within 20 min of arriving at the ED or within 60 min of emergency calls, they would be considered crossover and excluded from per-protocol analysis.

Subgroup analysis will be undertaken to evaluate the treatment effect in subgroups of patients defined by.

- age (> 50 vs ≤ 50 years): Older age is associated with worse OHCA outcomes; treatment benefit may differ by age.
- Gender: Biological and healthcare access differences may influence ECPR outcomes
- Time from emergency call to ED arrival (< 30 min, 30–45 min, vs 45–60 min): Low-flow time is critical for neurological outcomes; treatment effect may be time-dependent
- ST-segment elevation on ECG: Marker of acute coronary occlusion; patients with STEMI may benefit more from rapid reperfusion
- Initial lactate level (< 10 vs ≥ 10 mmol/L): Marker of metabolic derangement and tissue hypoperfusion; may predict treatment responsiveness
- Initial pH (< 7.0 vs ≥ 7.0): Marker of severe acidosis; extreme acidosis may limit treatment benefit.

### Cost-effectiveness analysis

A comprehensive cost-effectiveness analysis will be conducted from both healthcare system and societal perspectives. Data collection includes: (1) Direct medical costs**:** ECMO equipment and disposables, catheterization laboratory procedures, ICU and hospital stay, medications, rehabilitation services, and (2) Indirect costs: Lost productivity, caregiver burden (collected via structured surveys). The Outcomes of Quality-adjusted life years (QALYs) are calculated using EQ-5D-5L assessments at discharge, 3 months, 6 months, and 1 year.

Incremental cost-effectiveness ratio (ICER) will be calculated as cost per QALY gained. Sensitivity analyses will examine uncertainty in cost and outcome estimates. A willingness-to-pay threshold consistent with Taiwan's healthcare context (approximately $50,000–100,000 USD per QALY) will be used for interpretation."

### Ethical considerations

According to the Taiwan Medical Care Act, Chapter 4, Article 63 and Article 64 (latest version, June 28, 2023), written informed consent about the reason for the procedure, success rate, risk, and possible side effects of surgical operation and invasive intervention treatment must be obtained from the patient or, when underage or incapacitated, from a legal representative, spouse, or related relative. However, the provisions do not apply to medical emergencies. Informed consent could be waived for enrollment of eligible subjects in the critical emergency. Instead, a deferred consent will be sought whenever the emergency condition is under control. In ethical consideration, the study is conducted in accordance with the Declaration of Helsinki and is approved by the Institutional Review Board on the ethics of human investigation of each participating hospital.

## Discussion

Successful resuscitation of individuals experiencing OHCA requires a coordinated set of timely actions termed the “Chain of”Survival”—early recognition and activation of the emergency response system, early CPR, rapid defibrillation, and early advanced life support [22]. Our country has developed a well-organized EMS system, launching a series of optimization initiatives over the past decades, including prehospital 12-lead electrocardiography in 2012, public-access defibrillators in 2013, and dispatcher-assisted CPR implementation in 2013 [23]. According to a chronological, community-wide observational study in Taipei, 12.6% patients with non-traumatic OHCA initially presented with a shockable rhythm. With sequential implementations of optimizing initiatives, the rate of bystander CPR significantly increased from 32.6% in 2010 to 62.8% in 2017 (P < 0.001) [19]. Furthermore, each participating institution in this trial is capable of 24/7 ECMO cannulation and transportation, cardiac catheterization, and cardiac surgery, performing > 10 ECPR procedures per year. Expedited application of ECPR is only possible under highly controlled coordination and well-balanced strengthening of each link in the Chain of Survival. Considering the estimated distance from ECPR centers and EMS response times, we adopt the’load and go’ ECPR strategy for refractory shockable OHCA in this trial [24]. Because of the conflicting results from the 3 RCTs, critical appraisals of individual studies have addressed the differences in the study designs and offered insightful interpretations of the conclusions [11–13, 24]. Therefore, our study is designed to address the key disputable issues of the 3 RCTs, aiming to provide complementary data to support the use of ECPR for refractory shockable OHCA and to evaluate the cost-effectiveness of expedited application of ECPR (Table 3). We also extend the duration of call to ED to 60 min, thus it may lead to longer low flow time.

**Table 3:** Study characteristics of published randomized controlled trials and the ECLS-OHCA trial

	**ARREST**^**11, 17**^	**Prague OHCA**^**12, 17**^	**INCEPTION**^**13, 17**^	**ECLS-OHCA**
Refractory OHCA	Three defibrillation shocks	5 min of advanced cardiac life support	15 min of advanced life support	Cardiac arrest on arrival at ED
Witnessed with bystander CPR	±	+	+	+
Initial rhythm	VF or VT	All rhythms	VF or VT	VF or VT
Intervention	ECPR and early revascularization	Invasive bundle	ECPR	Expedited ECPR and early revascularization
Comparator	CCPR and early revascularization	CCPR with encouraged immediate invasive assessment	CCPR	CCPR with rescue ECLS and early revascularization
Randomization	In-hospital	Prehospital	Prehospital	In-hospital
Primary endpoint	Survival to hospital discharge	180-day neurological favorable survival	30-day neurological favorable survival	30-day neurological favorable survival
EMS response time (minutes, mean ± SD)	6.5 ± 2.4	8.9 ± 3.0	8 ± 4	6.4 ± 3.3 (estimate)
Time from emergency calls to ECLS (minutes, mean ± SD)	59 ± 28	62.0 ± 11.3	74.7 ± 18.2	≤ 45 (estimate), within 60 min is allowed
Time from randomization to ECLS initiation (minutes, mean ± SD)	12 ± 6	NR	NR	≤ 15 (estimate)

CCPR, conventional cardiopulmonary resuscitation; CPR, cardiopulmonary resuscitation; ECLS, extracorporeal life support; ECPR, extracorporeal cardiopulmonary resuscitation; ED, emergency department; EMS, emergency medical service; NR, not reported; OHCA, out-of-hospital cardiac arrest; SD, standard deviation; VF, ventricular fibrillation; VT, ventricular tachycardia

Outcomes of OHCA would not be optimized without addressing treatment of underlying causes and post-cardiac arrest care. Because acute coronary syndrome (ACS) is the most common reversible underlying cause in patients with shockable OHCA, as might be anticipated, patients with refractory shockable OHCA are more likely to be associated with more severe coronary lesions, e.g. left main involvement and acute occlusion of left anterior descending artery [25]. Due to the similarly time-sensitive nature of ischemic myocardial injury, early reperfusion is recommended in patients with ACS. Immediate coronary angiography (CAG) and primary percutaneous coronary intervention (PCI) after cardiac arrest is reasonable for patients with shock, electrical instability, signs of significant ongoing myocardial damage, or ongoing ischemia [14]. In patients with refractory shockable OHCA, ECMO-assisted intra-arrest PCI without continuing chest compressions has been proved feasible [26]. In high-volume ECMO centers, a treatment algorithm of ECPR-facilitated PCI for refractory shockable OHCA has been advocated. The Minnesota Resuscitation Consortium incorporated ECMO for hemodynamic stabilization and emergent PCI for coronary reperfusion in the clinical pathway of refractory shockable OHCA and reported improved hospital survival and survival with good neurological outcome of 55% and 50%, respectively [27].

When peripheral ECLS is used in cases with poor LV function, the increased LV afterload owing to the retrograde flow can lead to LV distension, impaired coronary perfusion, arrhythmias, pulmonary congestion, LV thrombus formation, etc., which might compromise the myocardial recovery [28]. Research has indicated that early active LV unloading during ECMO support improves survival in patients with cardiogenic shock [29]. Myocardial stunning frequently occurs after cardiac arrest. For patients with initial shockable rhythm, myocardial dysfunction may be further aggravated by the presence of acute coronary syndrome [30].

Concomitant LV unloading with ECPR was reported to be associated with favorable outcomes in patients with refractory cardiac arrest [31]. Practical guidelines for the post-cardiac arrest care in ECPR patients have incorporated LV unloading as one of the focused topics [32]. While none of the 3 RCTs had discussed their managements of LV distension complication in ECPR patients, our study will adopt novel LV unloading strategies under prespecified selection criteria.

## Conclusion

The ECLS-OHCA trial is a multicenter, open-label, interventional randomized clinical trial conducted in a metropolitan area with a well-developed EMS system and densely distributed healthcare facilities. All participating institutions are expert ECMO centers capable of 24/7 ECMO cannulation and transportation, allowing for expedited application of ECPR in eligible candidates with refractory shockable OHCA. Patients without identified noncardiac causes of OHCA will receive immediate CAG and primary PCI if indicated. Institutional protocols of post-cardiac arrest care will adhere to established recommendations from the latest guidelines. Active LV unloading is suggested in cases demonstrating the signs of LV overload. Our trial aims to provide complementary evidence supporting the use of ECPR in refractory shockable OHCA and to assess the cost-effectiveness of its early implementation.

## Supplementary Information

Supplementary Material 1.

Supplementary Material 2.

## Data Availability

No datasets were generated or analysed during the current study.
