# Supplementary material for: Emergency total extracorporeal life support versus standard advanced cardiac life support with rescue extracorporeal membrane oxygenation for refractory out-of-hospital cardiac arrest: protocol for the ECLS-OHCA randomized trial
Source: Scand J Trauma Resusc Emerg Med. 2026 Feb 3;34:53. doi: 10.1186/s13049-026-01557-w (PMC12958758; doi:10.1186/s13049-026-01557-w)
Supplement: Supplementary file 1 — Supplementary Material 1. [file 13049_2026_1557_MOESM1_ESM.docx]

**Intention-to-treat analysis**

Excluded (n= )

♦  Not meeting inclusion criteria (n= )

♦  Decline to participate (n= )

♦  Sustained ROSC (n= )

♦  Other reasons (n= )

## Follow-Up

Allocated to standard ACLS (n= )

♦ Received allocated intervention (n= )

♦ Did not receive allocated intervention

- Death (n= )
- ROSC >15 min (n= )
- ECPR by request (n= )

Allocated to emergency ECLS (n= )

♦ Received allocated intervention (n= )

♦ Did not receive allocated intervention

- Death (n= )
- ROSC >15 min (n= )
- Logistical failure to start ECPR (n= )

Lost to follow-up (n= )

Decline to participate (n= )

Lost to follow-up (n= )

Decline to participate (n= )

## Analysis

Analysed (n= )
♦ Excluded from analysis (n= )

Analysed (n= )
♦ Excluded from analysis (n= )

## Enrollment

## Allocation

Randomized (n= )

Assessed for eligibility (n= )
